# Universal vs. ASCO guidelines-based germline genetic testing for newly diagnosed breast cancer patients in resource-restricted settings

**DOI:** 10.3389/or.2025.1638255

**Published:** 2026-01-06

**Authors:** Hikmat Abdel-Razeq, Faris Tamimi, Sarah Abdel-Razeq, Baha Sharaf, Hanan Khalil, Hira Bani Hani, Hala Abu-Jaish, Suhaib Khater, Lulwa El Saket, Tamer Al-Batsh, Marwa Sh Abrahim, Mohammad Sammour, Asem Mansour

**Affiliations:** 1 Section of Hematology and Medical Oncology, Department of Internal Medicine, King Hussein Cancer Center, Amman, Jordan; 2 Department of Internal Medicine, School of Medicine, The University of Jordan, Amman, Jordan; 3 Department of Radiology, King Hussein Cancer Center, Amman, Jordan

**Keywords:** germline genetic testing, GGT, breast cancer, BRCA1, BRCA2, resource-restrictedcountries, personalized medicine

## Abstract

**Background:**

A significant subset of breast cancer cases is attributable to inherited pathogenic genetic variants. Germline genetic testing (GGT), particularly for *BRCA1* and *BRCA2*, represents a critical tool for precision oncology, enabling individualized risk stratification and the development of tailored therapeutic strategies.

**Methods:**

Consecutive newly diagnosed breast cancer patients eligible for GGT testing according to the latest American Society of Clinical Oncology (ASCO) guidelines were enrolled.

**Results:**

During the study period, 1,570 patients were enrolled, median age 51 (22-96) years, majority (n = 1,352, 86.1%) were Jordanian. Based on age criteria, 1,346 (85.7%) patients were eligible for testing. Another 134 (8.5%) were found eligible for testing because of other indications including personal or family history of breast and other cancers (n = 121, 7.7%), triple-negative disease (n = 9, 0.57%) and male gender (n = 4, 0.25%). In total, 1,480 (94.3%) patients were eligible for GGT as per ASCO guidelines, leaving only 90 (5.7%) patients not candidates for testing. Pathogenic/likely pathogenic variants were identified in 23 (7.8%) patients.

**Conclusion:**

Applying universal GGT for all newly diagnosed breast cancer patients, regardless of their age or risk factors, would slightly increase the pool of eligible patients, the burden of which can be justified given its impact on improving referral rate.

## 1 Introduction

Breast cancer continues to be the most common cancer diagnosed among women worldwide (1,2). Median age at diagnosis tends to be significantly lower in low-resource countries compared to Western societies (3,4). Almost half of breast cancer patients, in countries like Jordan, are diagnosed at age 50 years or younger, while only a small proportion are diagnosed after the age of 65 (5).

Pathogenic/likely pathogenic (P/LP) germline variants (PGVs) in cancer-predisposing genes, mostly in *BRCA1* and *BRCA2* have been linked to the etiology of breast and many other cancers. Identification of individuals with PGVs may reduce cancer burden on patients themselves, whereas at-risk relatives may benefit from intensive screening and risk-reducing strategies (6).

The percentage of breast cancer patients that are positive for *BRCA1* or *BRCA2* mutations varies depending on strategy utilized and the population tested. In unselected breast cancer cohorts, the prevalence of *BRCA1* and *BRCA2* P/LP variants is generally reported to be between 1.8% and 2.6% for *BRCA1* and 1.3%–2.1% for *BRCA2* (7–9). It is important to note that these mutations are more prevalent in certain subgroups, such as those with a family history of breast or ovarian cancer, in those with specific type of breast cancer like triple-negative disease (10), and in specific populations such as Ashkenazi Jewish women, where the prevalence can be higher (11, 12).

The wider adoption of multigene panel testing resulted in identifying other variants, some like *PALB2* are associated with an increased risk of breast cancer and may influence decisions regarding risk-reducing strategies and surveillance. Other mutations in genes like *CHEK2*, *BARD1*, *ATM*, *RAD51C*, *RAD51D* are linked to breast and other cancers (13, 14).

Patients are undergoing germline genetic testing based on international guidelines, including the National Comprehensive Cancer Network (NCCN) (15) and the American Society of Clinical Oncology (ASCO) (8, 16). These guidelines are frequently updated, and not all physicians, including medical and surgical oncologists, are familiar with such very frequent updates; a factor that may contribute to the lower referral of eligible patients for testing and counseling. Over the past few years, the age at which the NCCN recommends commence testing, regardless of personal or family history of cancer, was raised from 40 years to 45, then 50, and more recently was raised to 65 by the ASCO and the Society of Surgical Oncology (SSO). However, some professional societies, such as The American Society of Breast Surgeons, advocate universal testing of all women with breast cancer regardless of their age or risk factors. Such new direction is supported by several recent publications that showed higher rates of missed opportunities, should we restrict testing to those suggested by the guidelines (17, 18).

Universal germline genetic testing (GGT) via multigene panels (MGP) is not new in the field of cancer care, such practice is currently recommended for patients with ovarian, pancreatic, and metastatic prostate cancers (15).

The complexity of current guidelines, their frequent updates, the lack of dedicated cancer genetics programs, and the shortage of genetic counselors may all create barriers that limit access to genetic testing for eligible cancer patients (19). In this paper, we are testing the feasibility of universal testing of all newly diagnosed breast cancer patients regardless of their age, personal or family history of cancer in a resource-restricted setting.

## 2 Materials and methods

Study Design: This is a retrospective cohort study aimed to evaluate the implementation of universal GGT for all patients diagnosed with breast cancer in a resource restricted setting. The study was conducted at King Hussein Cancer Center (KHCC) and was approved by the Institutional Review Board (IRB).

Study Population: All consecutive adult patients, aged 18 and older, newly diagnosed with breast cancer were enrolled between 1 January 2023, and 30 April 2024. Data collection was completed by 15 May 2024, which served as the cutoff date for analysis. Patients with both invasive and non-invasive breast cancer diagnoses, including ductal carcinoma *in situ* (DCIS), were eligible. Exclusion criteria include patients who have previously undergone genetic testing for hereditary cancer syndromes. Given the retrospective nature of the study and lack of identifier, informed consent was waived for study enrollment, but all patients have consented for GGT at time of testing. The study adhered to ethical guidelines for human research. All data were de-identified and patient confidentiality was strictly maintained.

Germline Genetic Testing: Participants eligible for testing as per the NCCN or ASCO guidelines underwent GGT through next-generation sequencing (NGS) panels that assess known breast cancer-associated genes, including *BRCA1*, *BRCA2*, *TP53, CHEK2*, *PALB2*, and *ATM*. Testing was performed at an international reference laboratory using a validated, clinically recognized testing platform, and results were returned within two to 4 weeks. Testing was performed on DNA extracted from peripheral blood samples collected within 4 weeks of breast cancer diagnosis. A subset of patients’ relatives with P/LP variants were offered cascade testing after appropriate counseling and were not included in this cohort. A flowchart outlining the genetic testing pathway is provided in Supplementary Figure S2.

Clinical Data Collection: Demographic and clinical data were collected at baseline, including age, sex, nationality, family history of cancer, and tumor pathology. For patients who underwent genetic testing, we also collected data on the guidelines utilized for eligibility (either NCCN or ASCO criteria).

Data Analysis: The primary outcome of the study is to assess the proportion of breast cancer patients who are eligible for GGT as per the latest ASCO guidelines. Secondary outcomes include rates of P/LP variants in patients who underwent genetic testing, comparing those based on NCCN eligibility criteria versus ASCO eligibility criteria. Additionally, age-stratified analysis of P/LP variant frequency was performed. Descriptive statistics were used to summarize demographic and clinical characteristics, and Chi-square tests were used to compare rate of P/LP variants reported outcomes between different genetic test result eligibility.

## 3 Results

**FIGURE 1:**
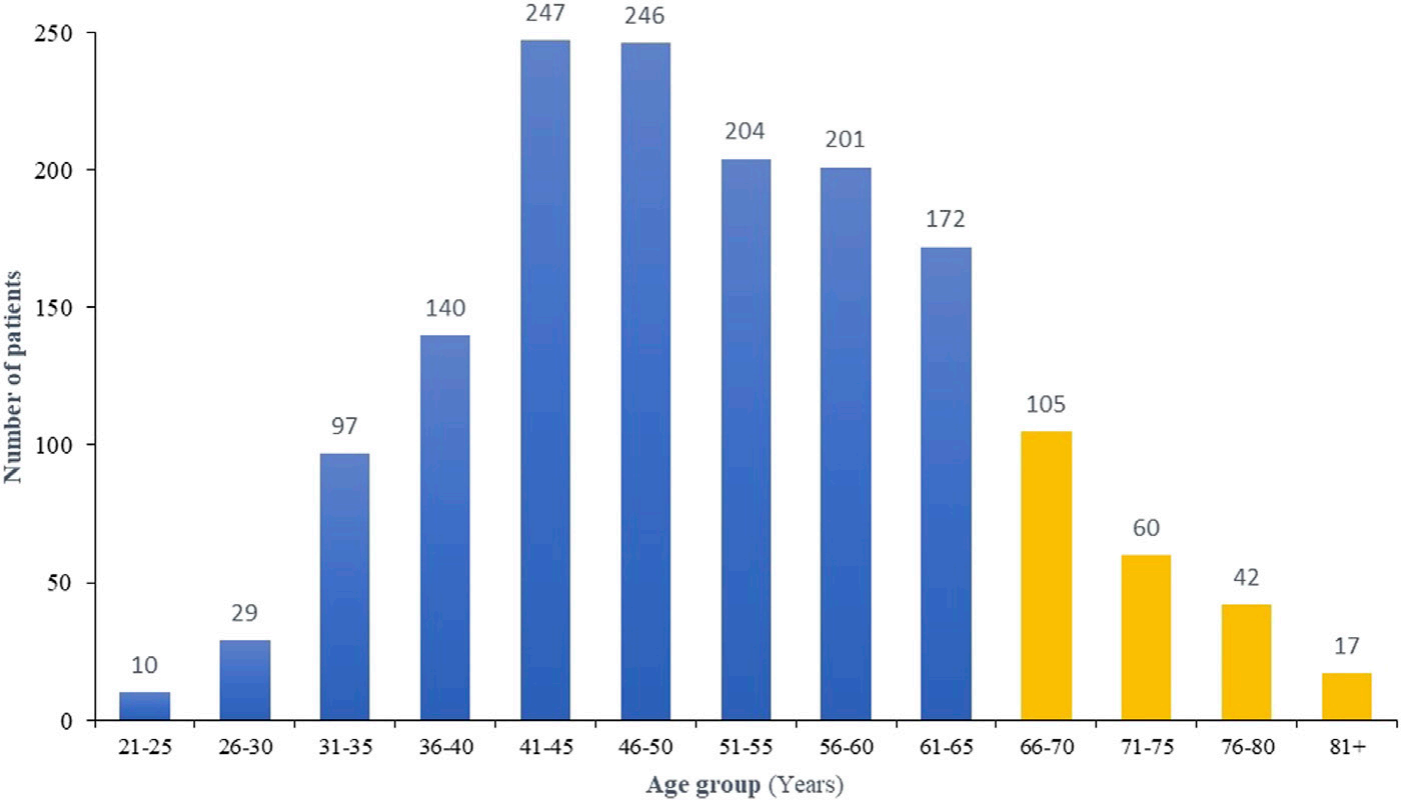
Age distribution of enrolled patients.

During the study period, a total of 1,570 patients were diagnosed with breast cancer, median age 51 (22-96) years with the majority (n = 1,346, 85.7%) of the patients were 65 years or younger. The distribution of patients’ age groups is illustrated in Figure 1.

Except for 11 (0.7%), all were female. Majority (n = 1,352, 86.1%) were Jordanian, while the rest (n = 218, 13.9%) were Arabs from neighboring countries or residing in Jordan. Invasive ductal carcinoma (IDC) was the most common pathology encountered in 1,253 (79.8%), while 150 (9.6%) had invasive lobular carcinoma (ILC) and 102 (6.5%) had ductal carcinoma *in situ* (DCIS), Table 1.

**TABLE 1:** Patients’ characteristics (n = 1,570).

Characteristic	n (%)
Age (years)	
Median (range)	51 (22–96)
≤65 years	1,346 (85.7%)
>65 years	224 (14.3%)
Sex	
Female	1,559 (99.3%)
Male	11 (0.7%)
Nationality	
Jordanian	1,352 (86.1%)
Other nationalities	218 (13.9%)
Histopathology	
Invasive ductal carcinoma (IDC)	1,253 (79.8%)
Invasive lobular carcinoma (ILC)	150 (9.6%)
Ductal carcinoma *in situ* (DCIS)	102 (6.5%)
Others	65 (4.1%)
Genetic testing	
Performed	1,142 (72.7%)
Not performed	428 (27.3%)
Eligibility assessment guidelines	
ASCO 2024	309 (19.7%)
NCCN	1,261 (80.3%)

IDC, invasive ductal carcinoma; ILC, invasive lobular carcinoma; DCIS, ductal carcinoma *in Situ*; NCCN, national comprehensive cancer network; ASCO, american society of clinical oncology.

Among the 1,570 patients, 1,142 (72.7%) underwent genetic testing. Genetic testing eligibility was assessed using NCCN guidelines for 1,261 (80.3%) patients diagnosed in January 2024 or earlier, while the updated 2024 ASCO guidelines were applied to 309 (19.7%) patients thereafter. Among those assessed using ASCO guidelines, 293 (94.8%) patients were eligible for genetic testing, compared to 849 (67.3%) patients using NCCN guidelines (p < 0.001). Among the 1,142 eligible patients who underwent genetic testing, P/LP variants were identified in 23 patients (7.8%) in the ASCO group and 77 patients (9.1%) in the NCCN group (p = 0.524), Table 2.

**TABLE 2:** ASCO guidelines-based versus NCCN guidelines-based germline genetic testing.

Variable	ASCO (n = 309)	NCCN (n = 1,261)	p value
Eligibility			
Eligible	293 (94.8%)	849 (67.3%)	<0.001**
Not eligible	16 (5.2%)	412 (32.7%)	
Test results among eligible#			
Negative	270 (92.2%)	772 (90.9%)	0.524
Pathogenic/likely pathogenic variants	23 (7.8%)	77 (9.1%)	

* Genetic testing eligibility was assessed using NCCN, guidelines for 1,261 patients diagnosed in January 2024 or earlier, while the updated 2024 ASCO, guidelines were applied to 309 patients thereafter.

^**^ Pearson’s Chi-squared test.

^#^ 1142 eligible patients.

We reviewed all patients, regardless of the eligibility criteria originally used, and reassessed their eligibility based on ASCO guidelines. Based on age criteria (i.e., patients aged ≤65 years), a total of 1,346 (85.7%) patients were eligible for testing. Among the other 224 (14.3%), medical records were reviewed for indications, other than age at diagnosis, for GGT. Another 134 (8.5%) were found to be eligible for testing because of personal or family history of breast and other cancers (n = 121, 7.7%), diagnosed with triple-negative disease (n = 9, 0.57%) and male gender (n = 4, 0.25%). In total, 1,480 (94.3%) patients were eligible for GGT as per the most recent ASCO guidelines, leaving only 90 (5.7%) who were not candidates for testing as per current guidelines, Table 3. When stratified by age, the P/LP variants was significantly higher among younger patients. Variants were identified in 13.9% of patients aged ≤39 years, compared with 8.0% in those aged 40–65 years and 4.0% in those older than 65 (p = 0.005) (Supplementary Table S4).

**TABLE 3:** Indications for germline genetic testing (n = 1,570).

Indication	Number (n)	Percentage (%)	Cumulative n (%)
Age ≤65 years	1,346	85.7	1,346 (85.7)
Family history of cancer	121	7.7	1,467 (93.4)
Triple-negative breast cancer	9	0.57	1,476 (94.0)
Male breast cancer	4	0.25	1,480 (94.3)
Not eligible for testing	90	5.7	1,570 (100)

## 4 Discussion

The presence of *BRCA1* and *BRCA2* mutations in breast cancer patients has significant implications in treatment decisions, primarily due to the distinct biological characteristics of these tumors and their response to specific therapies. *BRCA1*/2 mutations are associated with increased sensitivity to DNA-damaging agents, particularly poly (ADP-ribose) polymerase (PARP) inhibitors such as olaparib and talazoparib, which have shown efficacy in improving progression-free survival (PFS) in patients with BRCA-mutated HER2-negative breast cancer, both in early (20–23) and advanced disease settings (24–26). The use of PARP inhibitors is supported by clinical guidelines and has been incorporated into treatment regimens for BRCA-mutated breast cancer (27). Data was updated recently with overall survival (OS) benefits, too (28). Platinum-based chemotherapies are also effective in BRCA-mutated breast cancer due to their mechanism of inducing DNA cross-links, which are particularly lethal to cells with impaired DNA repair capabilities (29). These agents are often used in the neoadjuvant setting to improve pathologic complete response rates (pCR), especially in triple-negative breast cancer (TNBC) with BRCA mutations (29, 30). Additionally, the identification of BRCA mutations has implications for surgical decision-making and risk-reduction strategies. Patients with these mutations may opt for risk-reducing surgeries, such as bilateral mastectomy or salpingo-oophorectomy, to mitigate the risk of secondary cancers (31).

Several international professional societies, like the NCCN, have established guidelines recommending GGT for high-risk breast cancer patients. Current guidelines are somewhat complicated for most healthcare workers to follow; a fact that may contribute to the lower-than-expected referral rates for GGT in such patients. Such poor referrals are not restricted to low-income countries. Researchers from California used pooled cross-sectional data from the National Health Interview Survey, eligible patients were women with a history of breast or ovarian cancer meeting select 2017 NCCN eligibility criteria based on age at diagnosis and family history. Of the 47,218 women enrolled, 2.7% had a history of breast cancer. Over a third (35.6%) were eligible for GGT as per the NCCN criteria; of those, 29.0% reported a discussion about GGT with healthcare professionals, 20.2% were advised to undergo testing, and only 15.3% underwent genetic testing (32).

Various strategies have been explored to improve access and reduce delays in genetic testing. Universal genetic testing, in which germline genetic testing is offered to all patients diagnosed with breast cancer, regardless of their age at diagnosis or family history, represents an important step forward. However, challenges to adopt this approach including the cost, resource allocation, interpretation complexities, and ethical concerns, such as incidental findings (33). While NCCN recommends universal testing for epithelial ovarian, metastatic prostate, and exocrine pancreatic cancers, it is not recommended for breast cancer (34, 35).

The new ASCO–Society of Surgical Oncology Guideline recommended testing all newly diagnosed patients with breast cancer for *BRCA1*/2 mutation ≤65 years and select patients >65 years based on personal history, family history. This approach is not complex and can be implemented either through conventional genetic clinics or mainstream medical practices (8).

The median age of breast cancer diagnosis in Western countries, such as the United States (US) and Europe, is generally around 63 years. However, the median age at diagnosis in resource-restricted countries is at least a decade earlier (36–38). The variation in median age at diagnosis across different regions can be attributed to differences in population demographics, genetic factors, and lifestyle influences (39, 40). Jordan population is a younger one, with over 95% of the population are below the age of 65. Our study indicates that 85% of newly diagnosed breast cancer patients in countries like ours are eligible for testing just by age criteria (ASCO guidelines), Figure 1 (5). A proportion of older patients (>65 years) are eligible because of family or personally history of cancer, triple-negative disease or male gender, bringing the total to almost 95%.

One of the primary obstacles to the widespread adoption of universal testing in low- or middle-income countries (LMICs) is the high cost of genetic tests that can be prohibitively expensive for both patients and healthcare systems. Furthermore, the cost of follow-up care, including genetic counseling, cascade testing of close relatives and subsequent treatment, adds another layer of financial burden for patients in these countries.

Several studies have attempted to assess the cost-effectiveness of universal approach, mostly in Western healthcare systems. One study, conducted in the United Kingdom (UK) and the US, attempted to estimate the cost-effectiveness of multigene universal testing of all patients with breast cancer compared with the current practice of genetic testing based on family history or clinical criteria (guideline-based). All breast cancer patients in the universal testing group underwent *BRCA1*, *BRCA2* and *PALB2* testing. In the guideline-based group, only eligible patients underwent genetic testing. Cost analysis factored in contralateral preventive mastectomy in patients with BRCA/*PALB2* P/LP variants, risk-reducing salpingo-oophorectomy (RRSO) in patients with *BRCA1*/2 mutation. Analysis also considered the cost of cascaded testing for relatives of affected patients along with associated risk-reducing strategies including surveillance with magnetic resonance imaging or mammography screening, chemoprevention, or risk-reducing mastectomy and RRSO. Authors concluded that universal multigene testing for all patients with breast cancer is extremely cost-effective compared with guideline-based testing for UK and US healthcare systems (41). Investigators reached similar conclusion when universal testing was studied on Canadian patients. However, a similar conclusion might not be reached when such a program is applied in resource-restricted countries (42).

The shortage of clinical geneticists and genetic counselors is another challenge which led to the adoption of mainstream cancer genetic testing which allows non-geneticist clinicians, such as oncologists and surgeons, to order genetic tests without needing a genetics counselor referral (43–46). However, inconsistencies in implementation and reduced testing rates arise due to complex and frequently updated guidelines and the necessity for specialized training in hereditary cancer risk assessment for non-geneticist clinicians.

Recent LMIC data show that unselected germline testing identifies a high frequency of actionable BRCA and other pathogenic variants, with more than half of carriers missed by traditional criteria (47). Large cohorts from Brazil and Mexico similarly demonstrate that many mutation carriers lack “high-risk” features, leading to substantial under-ascertainment when selective guidelines are applied (48). Economic analyses from upper-middle-income settings highlight additional affordability challenges, showing that while broader testing can be cost-effective in Upper MICs, implementation requires careful resource assessment and planning in LMIC (49).

Higher rate of variants of uncertain significance (VUS) is another challenge (50). Rates of VUS varies with population tested, significantly higher with expanding the indications for testing and the number of genes tested within a multigene panel (51). The management of patients with VUS primarily involves a cautious and informed approach, as these variants are not clinically actionable and should not alter medical management based on the VUS result alone. Patients should be informed that VUS may be reclassified over time, and periodic follow-up is necessary to reassess the variant’s classification (52).

## 5 Limitations

This study has several limitations. First, its retrospective, single-center design may introduce selection bias and limit the generalizability of the findings. Second, follow-up outcomes and cascade testing were not assessed, and with time, there is a possibility that some VUS may be reclassified, which could affect interpretation of results. Third, cost-effectiveness analysis was not performed because financial data were outside the scope of the IRB approval. These factors should be considered when interpreting the findings.

## 6 Conclusion

Applying universal germline genetic testing for newly diagnosed breast cancer, regardless of their age, disease characteristics, family or personal history of breast or other cancers (universal testing), is feasible with little increase in the pool of eligible patients by few percentage points; the burden of which can be justified given its impact on improving referral rate and minimize the dependence on genetic counselors.

## Data Availability

The raw data supporting the conclusions of this article will be made available by the authors, without undue reservation.
