# Supplementary material for: Universal vs. ASCO guidelines-based germline genetic testing for newly diagnosed breast cancer patients in resource-restricted settings
Source: Oncol Rev. 2026 Jan 6;19:1638255. doi: 10.3389/or.2025.1638255 (PMC12815839; doi:10.3389/or.2025.1638255)
Supplement: Supplementary file 1 [file DataSheet1.PDF]

Supplementary Table 4. Pathogenic/Likely Pathogenic Variant by age group:

Age Group (years)	N (%)	No P/LP	P/LP	p-value
≤39	245 (15.6)	186 (86.1)	30 (13.9)	
40–65	1101 (70.1)	760 (92.0)	66 (8.0)	
>65	224 (14.3)	96 (96.0)	4 (4.0)	
Total	1570 (100)	1042 (91.2)	100 (8.8)	0.005

Note: P/LP = pathogenic or likely pathogenic variants. Statistical comparison by Chi-square test.

Supplementary Figure 2: Genetic Testing Workflow

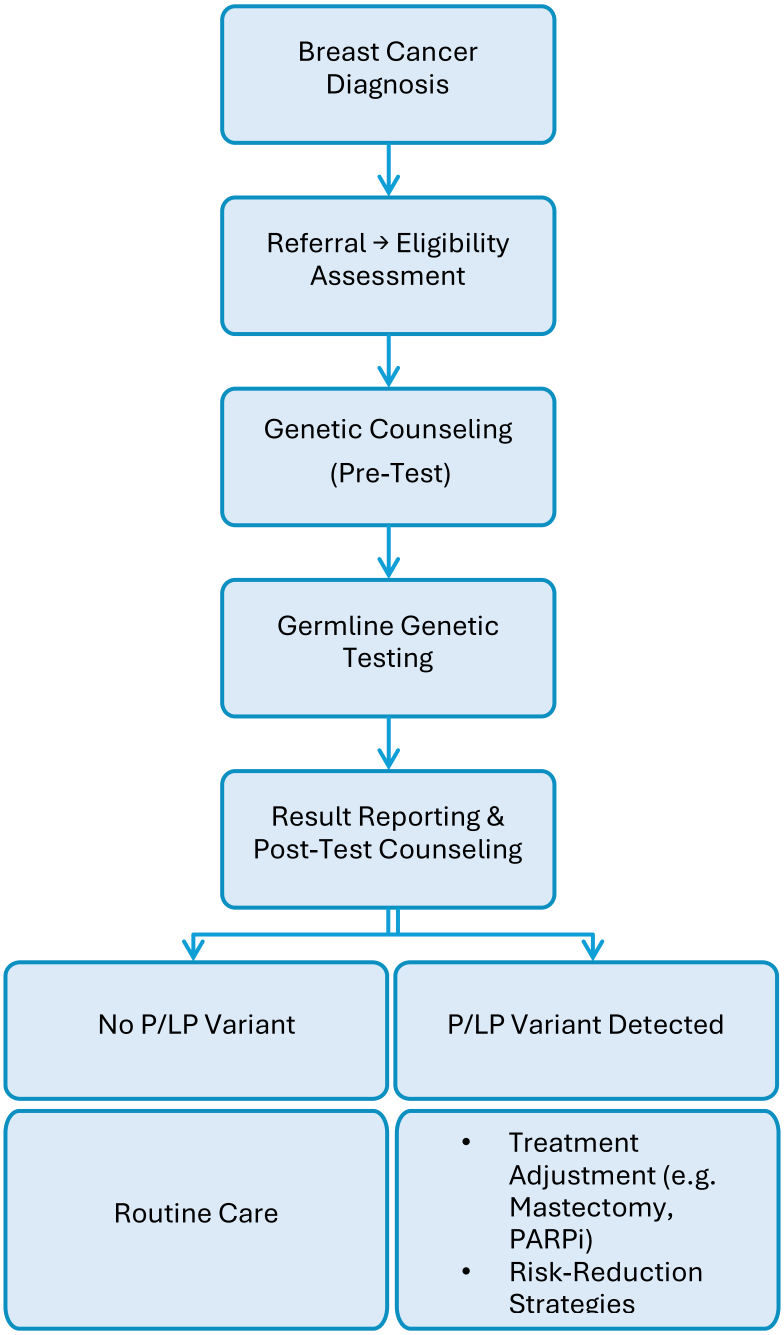

"This flowchart summarizes the implementation pathway for genetic testing in the study. The process begins with patient referral, followed by pre-test counseling, sample collection and genetic testing, and result reporting. The final step involves post-test counseling to guide clinical management based on test outcomes."
